# The risk of transmission of a viral haemorrhagic fever infection in a United Kingdom laboratory

**DOI:** 10.1371/journal.pntd.0005358

**Published:** 2017-05-18

**Authors:** Robert J. Shorten, Eleri Wilson-Davies

**Affiliations:** 1 Public Health Laboratory Manchester, Manchester Royal Infirmary, Manchester, United Kingdom; 2 University College London, Centre for Clinical Microbiology, Department of Infection, London, United Kingdom; 3 West of Scotland Specialist Virology Centre, Glasgow, United Kingdom; 4 Institute of Infection Immunity and Inflammation, University of Glasgow, Glasgow, United Kingdom; Banaras Hindu University (BHU), INDIA

The 2013–2016 Ebola virus outbreak centred in West Africa is the largest ever recorded and has resulted in a substantial global response across Guinea, Sierra Leone, and Liberia. The outbreak, which exceeds 28,500 cases [1], has seen sporadic cases exported to the United Kingdom, the United States, Spain, Switzerland, and Italy, either in the form of repatriation of confirmed cases or by case identification after travellers fell ill after returning from West Africa. Secondary transmission has been recorded in the US[2] and Spain.[3] The transmission of viral haemorrhagic fevers (VHFs) differs between aetiological agents. Ebola virus transmission between individuals occurs via direct contact with blood and body fluids[4]; nosocomial transmission is a prominent feature of outbreaks[5] but is not fully understood.[6]

There has, understandably, been a rise in suspected cases of Ebola virus disease (EVD) in travellers returning to the UK from affected areas. It is important to consider the probability of EVD or another VHF in a returning traveller alongside alternate differential diagnoses. The importation of VHFs into the UK is extremely rare and other infectious diseases, such as malaria or typhoid, are seen much more frequently. Clinicians will perform a risk assessment for such patients, focussing on the precise location of travel and exposure risks whilst there. All such patients require appropriate assessment and investigations whilst a diagnosis is made, and supportive pathology assays play a key part in this management. These investigations would be performed in pathology laboratories operating at Containment Level 2 (CL2), according to the UK Health and Safety Executive[7]. Such laboratories operate using good laboratory practice, which includes appropriate staff training, risk assessment, restricted access, and appropriate personal protective equipment (PPE), such as laboratory coats, gloves, and eye protection where applicable. In comparison, Containment Level 4 laboratories are complex facilities consisting of highly secure suites with restricted access, carefully controlled air-handling systems with filtered air, and the meticulous control and inactivation of hazardous waste. Manipulations of Hazard Group 4 pathogens[8] have traditionally been performed at this level of containment.

There have been 18 confirmed cases of VHF imported to the UK since 1971 (Public Health England data). Many of these cases presented to routine healthcare settings prior to the diagnosis being made. In most instances, pathology samples were obtained and processed in a routine manner within CL2 laboratories. Evidence from outbreaks strongly indicates that the main routes of transmission of VHF infection are direct contact (through broken skin or mucous membrane) with blood or body fluids, indirect contact with environments contaminated with splashes or droplets of blood or body fluids, and inoculation with sharps. Experts from the UK Advisory Committee on Dangerous Pathogens (ACDP) agree that there is no circumstantial or epidemiological evidence of an aerosol transmission risk from VHF patients.

A review of previous cases of VHFs imported into the UK shows that biochemistry, haematology, microbiology, and virology assays were performed using routine analysers in standard CL2 pathology laboratories. Often, these assays were performed prior to the diagnosis being made, yet no transmissions to laboratory workers were recorded (Table 1).[9][10][11][12] In addition, over 9,000 cases of Crimean Congo Haemorrhagic Fever (CCHF) were reported in Turkey between 2002 and 2014, with an estimated minimum 180,000 blood samples processed in routine laboratories with no additional precautions. A review was performed of 51 healthcare exposures that occurred in 9 centres where 4,869 of these patients were managed. Of these, only 2 cases in laboratory staff were identified. One may have been associated with a needle-stick injury and the other with handling samples while not wearing appropriate PPE (gloves).[13] There is no evidence of any risk of transmission when good laboratory practice is followed within a CL2 laboratory. Although it is reassuring that large numbers of samples from patients with CCHF infection have been processed safely in routine laboratories in Turkey, it should be noted that this bunyavirus is rarely transmitted person to person, so the parallels between this and other VHF viruses need to be carefully considered. The potential routes of transmission within a CL2 laboratory setting are, however, the same for all VHF agents.

**Table 1:** Details of staff exposure to confirmed cases of VHF imported into the United Kingdom.

Citation	Virus and Year	Location	Comments
Crowcroft NS et al. Journal of Infection (2004) 48, 221–228	Lassa 2000	UK	Patient initially admitted to Hospital for Tropical Diseases (HTD), University College London Hospitals (UCLH). 88 staff at the Royal Free Hospital (RFH) and UCLH followed up. No seroconversions.
Kitching A et al. Eurosurveillance 14(6), 12 February 2009	Lassa 2009	UK	Patient initially admitted to Homerton University Hospital. Many samples taken and processed for in-house and referred tests over 2 weeks. Transferred to HTD (UCLH) for further laboratory tests. Transferred to High Level Isolation Unit (HLIU) at RFH where patient passed away. 72 lab staff considered low risk (handled samples with personal protective equipment [PPE]). No secondary cases reported.
Atkin S et al. Eurosurveillance 14(10), 12 March 2009	Lassa 2009	UK	Admitted to HTD (UCLH). Passed away the same day. Laboratory staff: 21 “no risk”, 45 “low risk”, 3 “high risk”. No secondary cases reported.
Barr DA et al. Lancet. 2013 Oct 26	CCHF 2012	UK	Admitted via Glasgow Accident and Emergency department (A&E). Routine blood samples taken over 36 hours before diagnosis of CCHF confirmed and transferred to HLIU at RFH. No secondary cases reported.

Laboratory-acquired cases of EVD were reported in the West African outbreak; however, it should be remembered that initial laboratory work in this outbreak was performed with extremely limited facilities and resources. We are not aware of any imported cases of EVD to resource-rich settings that have resulted in laboratory transmission, even when samples were analysed with no additional precautions prior to diagnosis.

In vitro diagnostic systems (IVDS) include analysers that automate the diagnostic process in clinical laboratories. These in vitro diagnostic medical devices (IVDMD) therefore process blood or blood in suspension of testing fluids. Consequently, all IVDMD which perform assessments on patients’ blood will regularly be challenged by exposure to blood-borne viruses (BBV) which circulate within the population. Therefore, they should be designed to eliminate or reduce as far as reasonably practicable the risk of infection to users and other persons, which includes the staff who service the devices. Fundamental to this is the manufacturer’s design to minimise the leakage of fluid and contamination, which may lead to microbial exposure during normal use or servicing. [14]

Important viruses which are present in blood (viraemia) during chronic infections include BBVs such as human immunodeficiency virus (HIV), hepatitis B virus (HBV), and hepatitis C virus (HCV). Most viruses have the potential to cause a viraemia during an acute infection. High hazard pathogens which are present in blood during acute infections include Ebola, CCHF, Lassa fever, Marburg, dengue, chikungunya, Middle East Respiratory Syndrome coronavirus (MERS-CoV), and severe acute respiratory syndrome coronavirus (SARS-CoV).

**Fig 1:**
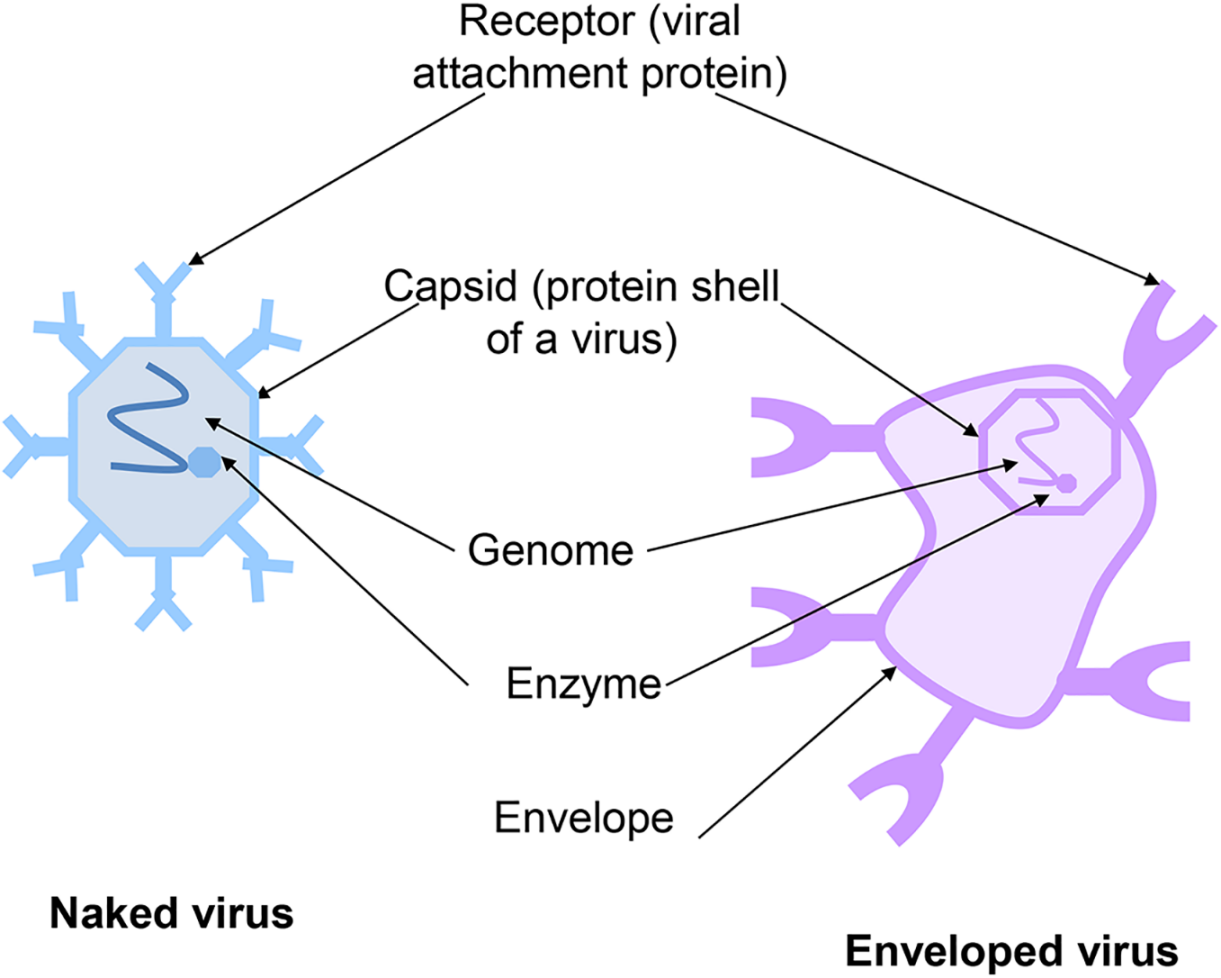
Schematic of an enveloped and non-enveloped virus.

**Fig 2:**
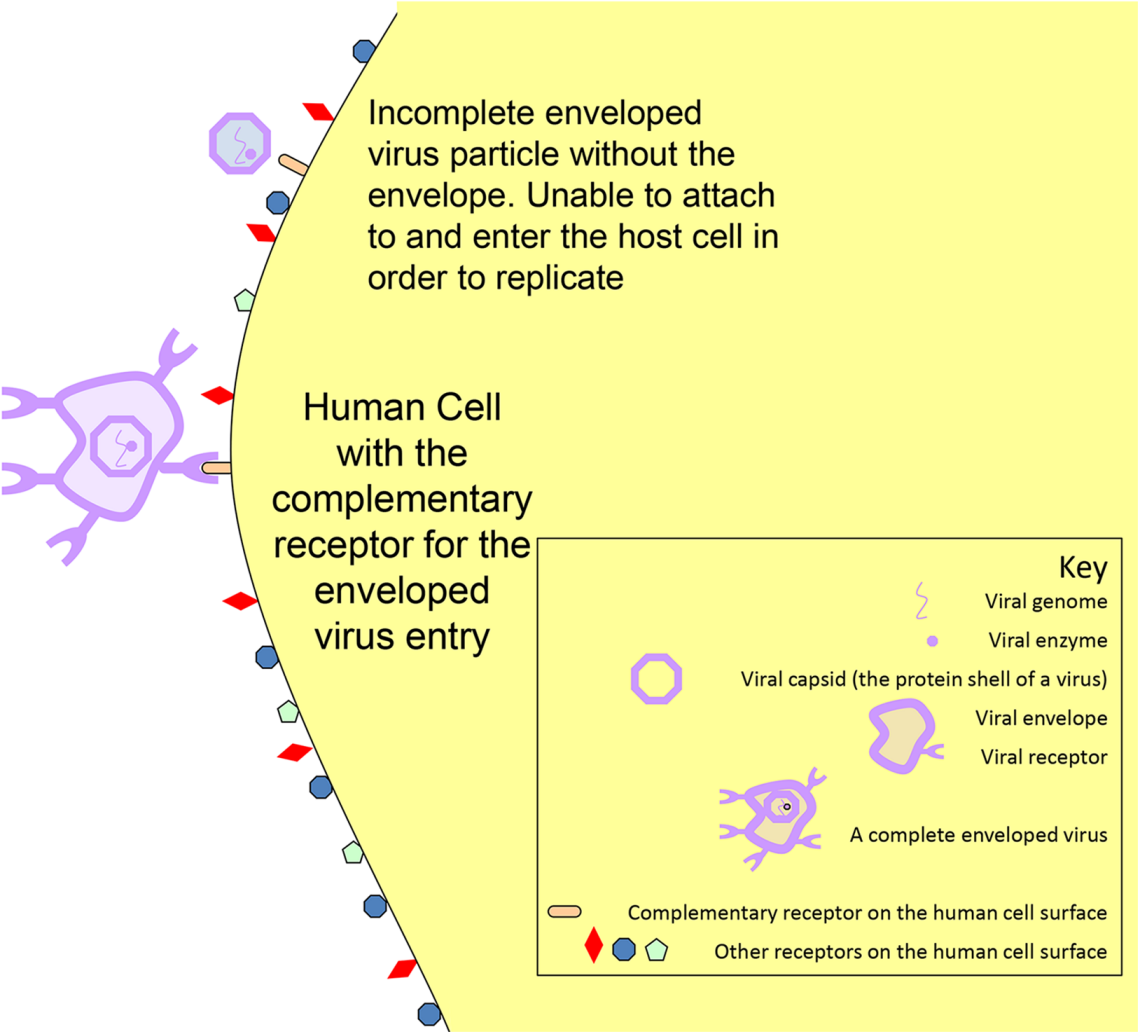
Schematic of viral attachment.

Viruses are either naked or enveloped in structure (Fig 1). The envelope (where present) is derived from the surrogate host cell, which, along with the associated viral receptors, is essential for attachment and host cell entry. Removal of the viral envelope inactivates the virus, preventing replication and subsequent host infection (Fig 2). All of the high hazard viral diseases cited are caused by enveloped viruses.

IVDS should be decontaminated prior to inspection, maintenance, repair, or disposal, either on site or at the manufacturer's or agent's premises.[15] Decontamination must be carried out in line with the manufacturer’s instructions using methods validated to inactivate enveloped viruses. The enveloped viruses are among the most susceptible pathogens to disinfectants. [16] This is due to the presence of the lipid envelope, which is compromised by most biocides. Compounds with validated efficacy against enveloped viruses include alcohols, aldehydes, biguanides (e.g., chlorhexidine), halogens, peroxygen compounds (e.g., hydrogen peroxide), peracetic acid, some phenols, and some quaternary ammonium compounds.

Manufacturers must validate their decontamination method against appropriate surrogate model enveloped viruses. Laboratory viruses vary between strains and wild-type viruses. Studies, by necessity, use laboratory strains which may be grown to high titre and efficiently assayed. Any virus used in a validation study is, therefore, a model virus. Viral inactivation validation studies have successfully used surrogate model viruses with properties similar to wild-type viruses to provide evidence for the safety of human blood plasma products for over a decade.[17] Studies using model viruses are also used for pharmaceuticals, derived from human and/or animal sources, including recombinant proteins produced in eukaryotic cell lines, vaccines, and some Class III medical devices.[18]

Viral inactivation validation studies must inactivate a range of enveloped viruses, using at least 3 viruses to represent different properties that IVDMDs are expected to be challenged against. A suitable model virus for HIV, HBV, and HCV must be included. If an enveloped virus has been shown to have a higher resistance to the manufacturer’s disinfectant class, then a model for that virus should also be included. Once dried on inanimate surfaces, viruses are less susceptible to decontamination than when hydrated in suspension.[19][20] Validation studies should therefore include a quantitative suspension test for the assessment of internal decontamination procedures [15] and quantitative carrier tests [21] or appropriate alternative methods. An effective and reliable decontamination method will show a reduction of 4 log^10^.[22] Despite some pathogens causing a very high titre viraemia, the design of IVDMDs reduces their contamination as far as reasonably practicable. The design of IVDMDs means they only require a small volume of blood to perform an analysis. Consequently, when an effective and reliable decontamination method is in place that provides a reduction of 4 log^10^, this will inactivate any residual enveloped virus within the IVDMD. Decontamination therefore renders the IVDMD safe to use and service after challenge with any enveloped virus. Once assessed by appropriately designed viral inactivation validation studies on relevant surrogate model enveloped viruses, the decontamination process has been shown to be effective against all known and future emerging enveloped viruses, which includes Ebola, CCHF, Lassa fever, and Marburg virus.

Guidance issued by the ACDP therefore states that samples taken from these patients may be safely processed using standard precautions, good laboratory practice, and PPE, as the risk of infection from these samples is low. [23] It also states that routine decontamination procedures are adequate in these situations. The guidance further explains that autoanalysers are the preferred method for processing such specimens. Sealed buckets should be used for any centrifugation procedures that are not undertaken within an autoanalyser. In certain circumstances, the use of discrete analysers may need to be considered, but these are not a safer option. Point of care (POC) blood gas analysers present a high risk of splashing and should only be used in exceptional circumstances with suitable barrier protection PPE for staff and in a controlled environment. When preparing blood film slides for malaria testing, consideration should be given to the potential for splash and therefore should be carried out in a microbiological safety cabinet or, alternatively, facial protection should be used. Likewise, blood cultures and blood cross-matching may be performed at CL2 following appropriate risk assessment.

We also know from Health & Safety Executive Reporting of Injuries, Diseases and Dangerous Occurrence Regulations (RIDDOR) data that rates of infection with BBVs in healthcare workers are low, and the majority of these are needle-stick associated and not in laboratory staff. [24] This is also supported by data from the UK significant occupational exposures surveillance system. [24] Tens of thousands of samples are processed daily in routine pathology laboratories that are, often unknown to us, positive for BBVs.

The important consideration here is about the clinical well-being and appropriate investigation and management of the patient. A delay in diagnosis of other traveller-associated infections, such as malaria or typhoid, as well as lack of access to supportive pathology assays, can be fatal. Laboratory staff welfare is equally important, and the application of good laboratory practice in a risk-assessment–led setting indicates that such samples may be analysed safely in routine CL2 laboratories.
